# Incidence and Outcomes of Occult Uterine Cancer in Uteri Removed for Prolapse

**DOI:** 10.1007/s00192-025-06380-y

**Published:** 2025-10-14

**Authors:** Morgan Gruner, Surabhi Tewari, Meng Yao M., Katie Propst, Mariam AlHilli

**Affiliations:** 1 Obstetrics, Gynecology, Women’s Health Institute, Cleveland Clinic, Desk A81, 9500 Euclid Avenue, Cleveland, OH 44195 USA; 2 Cleveland Clinic Lerner College of Medicine of Case Western Reserve University School of Medicine, Cleveland, OH 44195 USA; 3 Department of Qualitative Health Sciences, Cleveland Clinic, Cleveland, OH 44195 USA; 4 Division of Urogynecology, Department of Subspeciality Care for Women’s Health, Obstetrics and Gynecology Institute, Cleveland Clinic, Desk A81, 9500 Euclid Avenue, Cleveland, OH 44195 USA; 5 Division of Gynecologic Oncology, Department of Subspeciality Care for Women’s Health, Obstetrics and Gynecology Institute, Cleveland Clinic, 9500 Euclid Ave., Cleveland, Ohio 44195 USA

**Keywords:** Hysterectomy, Occult endometrial carcinoma, Pelvic organ prolapse, Uterine cancer

## Abstract

**Introduction and Hypothesis:**

To identify the incidence of occult endometrial cancer diagnosed following hysterectomy for the repair of pelvic organ prolapse, and compare patient outcomes to a control cohort of preoperatively diagnosed endometrial cancer patients.

**Methods:**

A retrospective cohort study of patients ≥50 years with endometrial cancer between 2010 and 2020 was performed. Demographic, clinical, surgical, and oncologic variables were compared between occult endometrial cancer and preoperatively diagnosed endometrial cancer cohorts.

**Results:**

One thousand seventy-two patients were included, of which 30 (2.8%) had occult endometrial cancer diagnosed after prolapse surgery and 1042 (97.2%) were in the preoperatively diagnosed historic cohort. The incidence of occult endometrial cancer was 0.56% for all hysterectomies performed for pelvic organ prolapse. Patients in the occult endometrial cancer cohort were more likely to have grade I disease (85.2% vs. 52.1%, *p* < 0.001), less likely to have lymphovascular space invasion (10.7% vs. 31.8%) or >50% myometrial invasion (11.1% vs. 24.0%, *p* = 0.004) compared to the preoperatively diagnosed patients. Ten occult endometrial cancer patients (33.3%) underwent a second staging procedure; 83.3% (*n* = 25) of patients received care in compliance with comprehensive national cancer guidelines. Five-year recurrence free survival was 95.0% (95% CI 85.4–100%) and 66.8% (95% CI 59.3–74.4%) for preoperatively diagnosed cohort and occult endometrial cancer cohort, respectively, while 5-year overall survival was 90.9% (95% CI 78.9–100%) and 83.0% (95% CI 75.5–90.5%), respectively.

**Conclusions:**

The rate of incidental endometrial cancer after hysterectomy for pelvic organ prolapse was 0.56% in our cohort. Most occult diagnosed patients are diagnosed with early-stage and low-grade disease. The majority received care concurrent with National Comprehensive Cancer Network recommendations.

## Introduction

Endometrial cancer (EC) is the most common gynecologic malignancy in women with approximately 66,200 estimated new cases diagnosed in 2023 and 13,030 expected deaths in the United States [1, 2]. The 5-year relative survival rate is 81.1%, and approximately 3.1% of women will be diagnosed with uterine cancer at some point during their lifetime [2]. Risk factors for EC include age, obesity, estrogen hormone replacement therapy, nulliparity, and a family history of EC [3].

A woman’s lifetime risk of undergoing a procedure for urinary incontinence or pelvic organ prolapse (POP) by the age of 80 is estimated to be 11% [4]. Analysis of the National Hospital Discharge Summary estimated that over 200,000 and 100,000 inpatient surgical procedures are performed in the United States annually for POP and female urinary incontinence, respectively [5, 6]. Prolapse is the most common indication for hysterectomy in women greater than 55 years of age [7]. The risk of an incidental finding of uterine pathology in women undergoing hysterectomy for prolapse is overall low, studies quote percentages ranging from 0.5–2.6% [8–10]. An incidental diagnosis of malignancy after surgery for prolapse or other benign indication can result in deviations from standard management that may potentially alter patients’ outcomes.

Knowing the incidence of uterine malignancy at the time of surgery for prolapse can help guide preoperative evaluations to identify patients at risk and direct preoperative counseling. Our study aim was to identify the incidence of occult endometrial cancer identified on pathology specimens from surgeries performed for POP at our institution. In addition, we sought to evaluate the oncologic outcomes of patients with an occult diagnosis of EC in comparison to women with a known preoperative diagnosis of EC.

## Methods

### Study Design

We performed an institutional review board approved, retrospective cohort study of patients aged 50 years and older who had an incidental diagnosis of EC during primary surgery for POP at a single, tertiary care institution between January 1, 2010 and December 31, 2020. Patients were excluded if hysterectomy of any route (vaginal, laparoscopic, or open) was not performed.

Patients were identified using Current Procedural Terminology (CPT) codes for prolapse surgery. This cohort was then cross-referenced with patients who had International Classification of Diseases-9 or −10 codes identifying endometrial malignancy. Procedures for POP repair for which CPT codes were used included colpopexy, uterosacral ligament suspension, paravaginal repair, colpocleisis, Le Forte colpocleisis, sacrospinous ligament suspension, anterior colporrhaphy, combined anterior/posterior colporrhaphy, posterior colporrhaphy, colpoperineorrhaphy, colpoperineorrhaphy with repair of rectocele, burch or Marshall–Marchetti–Krantz procedure (primary or repeat), laparoscopic burch or Marshall–Marchetti–Krantz, fascia or synthetic sling, or laparoscopic sling. Identified patients were reviewed by two independent persons to verify concordance with inclusion criteria. Most procedures were performed by a urogynecologist with a few completed by either a general OBGYN or urologist.

This cohort was compared to the preoperatively diagnosed group, who had a diagnosis of EC prior to hysterectomy and completed definitive surgery at the Cleveland Clinic Foundation between January 1, 2004 and August 1, 2016 as previously described by Son et al. [11]. Patients undergoing fertility sparing treatment were excluded.

### Data Collection

We queried the electronic medical record for patient, surgical, and oncologic parameters. All data was collected and stored in a secure REDCap database [12]. Patient characteristics, including age at primary surgery, race, body mass index, history of smoking, comorbidities, and parity were included.

Data specific to the prolapse surgery was collected, including hysterectomy approach and additional procedures performed. Oncologic parameters specific to the occult cohort collected included patient presentation at our institutional tumor board, and requirement for subsequent surgical staging. Time to a secondary surgical procedure was defined as days elapsed between the date of primary prolapse surgery and staging procedure. Optimal management of disease per the National Comprehensive Cancer Network (NCCN) guidelines for uterine cancer was noted [13].

Oncologic parameters collected for both the occult and preoperatively diagnosed cohorts included stage and grade at diagnosis, histology, lymphovascular space invasion (LVSI), tumor size, and depth of myometrial invasion (MMI). Adjuvant therapy, including chemotherapy, extended beam radiation therapy, and vaginal brachytherapy was documented.

### Statistical Analysis

Approximately normally distributed continuous measures were summarized using means and standard deviations and compared using two-sample *t*-tests. Continuous measures that demonstrated departure from normality and ordinal measures were summarized using medians and quartiles or frequencies and percentages and compared using Wilcoxon rank sum tests. Categorical factors were summarized using frequencies and percentages and were compared using Pearson’s chi-square tests or Fisher’s exact tests. Owing to the different follow-up durations between the two cohorts and the low event rates in the occult cohort, only descriptive survival analysis was done. Survival starting date was set to be the surgery date, and month was defined as 30 days. Data analysis was performed using SAS software (version 9.4; SAS Institute, Cary, NC). *P* values of < 0.05 were considered significant.

## Results

### Patient Demographics

A total of 1072 patients were included in this analysis of which 30 (2.8%) patients were in the occult cohort and 1042 (97.2%) were in the preoperatively diagnosed cohort. Patient characteristics comparing the two cohorts are illustrated in Table 1. The age at surgery was similar between the occult and preoperatively diagnosed cohorts (mean 67.2 years ± SD 9.1 vs. 63.9 years ± 9.2, *p* = 0.054). Patients in the occult cohort had a lower body mass index (kg/m^2^) in comparison to the preoperatively diagnosed cohort (30.1 ± 6.4 vs. 34.7 ± 9.3, *p* < 0.001) and higher rate of smoking history (40.0% vs. 0.29%, *p* <0.001). The distribution of race differed significantly between the two cohorts as the occult cohort had fewer African American patients (3.3% vs. 9.4%) and more Asian/Pacific Islander patients (6.7% vs. 1.9%) (*p* = 0.006) in comparison to the preoperatively diagnosed cohort. The two cohorts had similar rates of hypertension diabetes mellitus pulmonary disease, cardiac disease, other cancers, and Lynch syndrome (Table 1).

**Table 1:** Demographics comparing the occult endometrial cancer to preoperative endometrial cancer cohorts

		Occult (*N* = 30)		Preoperative (*N* = 1 042)		
Factor	Total (N=1,072)	*N*	Statistics	*N*	Statistics	*p* value
**Age at surgery**	64.0 ± 9.3	30	67.2 ± 9.1	1042	63.9 ± 9.2	0.054^a1^
**Body mass index (kg/m**^**2**^**)**	34.6 ± 9.3	30	30.1 ± 6.4	1031	34.7 ± 9.3	***<0.001***^***a2***^
**Race**		30		1024		***0.006***^***d***^
Non-Hispanic White	935 (88.7)		26 (86.7)		909 (88.8)	
Hispanic	1 (0.09)		1 (3.3)		0 (0.00)	
Black/African American	97 (9.2)		1 (3.3)		96 (9.4)	
Asian/Pacific Islander	21 (2.0)		2 (6.7)		19 (1.9)	
**Comorbidities**						
Hypertension	628 (58.6)	30	22 (73.3)	1042	606 (58.2)	0.096^c^
Diabetes	304 (28.4)	30	6 (20.0)	1042	298 (28.6)	0.30^c^
Pulmonary disease	174 (16.2)	30	3 (10.0)	1042	171 (16.4)	0.46^d^
Cardiac disease	229 (21.4)	30	4 (13.3)	1042	225 (21.6)	0.28^c^
Other cancer history	150 (14.1)	30	3 (10.0)	1032	147 (14.2)	0.79^d^
**History of Lynch syndrome**		30		1042		0.99^d^
Yes	5 (0.47)		0 (0.00)		5 (0.48)	
No	1,061 (99.0)		30 (100.0)		1031 (98.9)	
Unknown	6 (0.56)		0 (0.00)		6 (0.58)	
**Smoking history**	15 (1.4)	30	12 (40.0)	1042	3 (0.29)	***<0.001***^***d***^
**Parity**	2.0 [1.00, 3.0]	29	2.0 [2.0, 3.0]	993	2.0 [1.00, 3.0]	0.091^b^

Statistics presented as mean ± SD, median [P25, P75], *N* (column %)

*p* values: *a1* *t*-test, *a2* Satterthwaite *t*-test, *b* Wilcoxon Rank Sum test, *c* Pearson’s chi-square test, *d* Fisher’s Exact test

### Urogynecologic Surgery Parameters

A total of 5376 unique POP surgeries occurred between January 1, 2010 and December 30, 2020 at our institution. A total of 30 patients had an incidental diagnosis of EC confirmed postoperatively after prolapse surgery for an overall incidence of EC of 0.56%. No patients diagnosed with EC demonstrated signs or symptoms of EC preoperatively, particularly postmenopausal bleeding. The surgical parameters for the occult cohort are described in Table 2. Most patients had a vaginal hysterectomy (*n* = 22, 75.9%). Ten percent (*n* = 3) had a unilateral salpingectomy, 40.0% (*n* = 12) had a bilateral salpingectomy, 6.7% (*n* = 2) had a unilateral oophorectomy, 33.3% (*n* = 10) had a bilateral oophorectomy, and 3.3% (*n* = 1) had a colpocleisis. The median uterine weight for all hysterectomies performed in the occult cohort was 59.3 grams [Interquartile range (IQR) 42.5–99.0].

**Table 2:** Characteristics of surgical variables for occult endometrial cancer cohort

	Total (*N* = 30)	
Factor	*N*	Statistics
**Hysterectomy approach**	29	
Abdominal		2 (6.9)
Laparoscopic		5 (17.2)
Vaginal		22 (75.9)
**Type of urogynecology surgery**		
Tension free vaginal tape sling	30	5 (16.7)
Transobturator tape sling	30	7 (23.3)
Anterior colporrhaphy	30	21 (70.0)
Posterior colporrhaphy	30	18 (60.0)
Sacrocolpopexy	30	5 (16.7)
Uterosacral ligament suspension	30	16 (53.3)
Perineorrhaphy	30	5 (16.7)
Sacrospinous ligament suspension	30	2 (6.7)
Unilateral salpingectomy	30	3 (10.0)
Bilateral salpingectomy	30	12 (40.0)
Unilateral oophorectomy	30	2 (6.7)
Bilateral oophorectomy	30	10 (33.3)
Colpocleisis	30	1 (3.3)
**Uterus weight (g)**	28	59.3 [42.5, 99.0]
**Staging at urogynecology surgery**	30	0 (0)
**Adnexal involvement**	19	3 (15.8)
**Cervical involvement**	27	0 (0)
**Patient presented at tumor board**	30	4 (13.3)
**Second staging procedure required**	30	
Yes		10 (33.3)
No		12 (40.0)
Yes but not performed		8 (26.7)
**Time to second staging procedure (days)**	10	52.0 [28.0, 64.0]
**Compliance with NCCN guidelines**	30	25 (83.3)

*NCCN* National Comprehensive Cancer Network

Statistics presented as Median [P25, P75], *N* (column %).

Of the 30 patients diagnosed with occult EC, no patients underwent lymph node assessment during prolapse surgery, 13.3% (*n* = 4) of patients were presented at a multidisciplinary tumor board, and 33.3% (*n* = 10) underwent a second staging procedure. The median time to second staging procedure was 52.0 days [IQR 28.0–64.0]. In 26.7% (*n* = 8) patients, a second staging procedure was documented as discussed with the patient in the medical record but was deferred due to either patient preference or low risk status. For 40.0% (*n* = 12) of patients, a second staging procedure was not recommended. Adnexal involvement was present in 15.8% (*n* = 3) of patients who had an oophorectomy either at initial surgery or at secondary staging. Overall, 83.3% (n*n* = 25) of patients received care in compliance with NCCN guidelines for uterine cancer.

### Oncologic Parameters for Occult Endometrial Cancer Versus Preoperative Diagnosed Cohorts

**Fig. 1:**
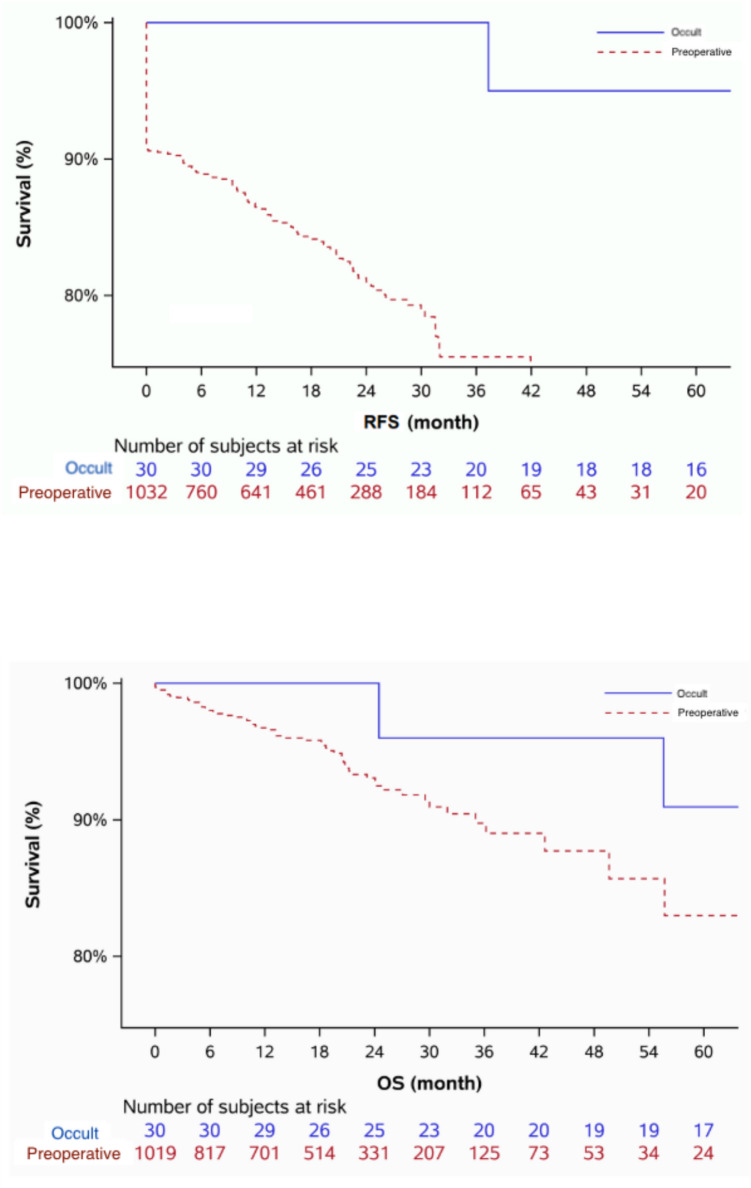
**A/B:** Oncologic outcomes in occult endometrial cancer versus preoperatively diagnosed endometrial cancer cohorts

There was no difference in stage or histology at diagnosis with the majority of patients presenting with stage I or II (87.5% vs. 83.7%, *p* = 0.78) and endometrioid histology (90.0% vs. 80.1%, *p* = 0.44) in the occult and preoperatively diagnosed cohorts, respectively (Table 3). Patients in the occult cohort were more likely to present with International Federation of Gynecology and Obstetrics (FIGO) grade 1 disease (85.2% vs. 52.1%, *p* < 0.001) when compared to the preoperatively diagnosed cohort and were less likely to have LVSI (10.7% vs. 31.8%, *p* = 0.017) or MMI of > 50% (11.1% vs. 24.0%, *p* = 0.004). Patients in the occult cohort had smaller median tumor sizes on pathology (2.0 cm IQR [0.60–2.7] vs. median 3.0 cm IQR [1.1–4.5], *p* = 0.005) in comparison to the control cohort and were less likely to receive any form of adjuvant therapy (24.1% vs 48.9%, *p* = 0.008), including chemotherapy (13.3% vs. 30.7%, *p* = 0.041) and vaginal brachytherapy (10.0% vs. 32.9%, *p* = 0.008). However, no differences in likelihood of receiving external beam radiotherapy between cohorts was noted (*p* = 0.11). With a median follow-up duration of 72.0 months [IQR 32.3–94.5], two recurrences (6.7%) and 5 deaths (16.7%) were observed in the occult cohort. As is seen in Fig. 1A/B, the 5-year recurrence free survival was 95.0% (95% CI 85.4% −100%) and 5-year overall survival was 90.9% (95% CI 78.9%–100%). The preoperatively diagnosed cohort had a median follow-up duration of 18.0 months [IQR 8.9–26.4] with 5-year recurrence-free survival of 66.8% (95% CI 59.3–74.4%), and 5-year overall survival of 83.0% (95% CI 75.5–90.5%).

**Table 3:** Oncologic variables comparing occult endometrial cancer to preoperative endometrial cancer cohorts

		Occult (*N*=30)		Preoperative (*N* = 1042)		
Factor	Total (*N*=1,072)	*N*	Statistics	*N*	Statistics	*p* value
**Stage at diagnosis**		24		1014		0.78^d^
I/II	870 (83.8)		21 (87.5)		849 (83.7)	
III/IV	168 (16.2)		3 (12.5)		165 (16.3)	
**Grade**		27		936		***<0.001***^***b***^
FIGO 1	511 (53.1)		23 (85.2)		488 (52.1)	
FIGO 2	272 (28.2)		4 (14.8)		268 (28.6)	
FIGO 3	180 (18.7)		0 (0.00)		180 (19.2)	
**Histology**		30		1,037		0.44^d^
Endometrioid	858 (80.4)		27 (90.0)		831 (80.1)	
Serous	77 (7.2)		1 (3.3)		76 (7.3)	
Clear cell	17 (1.6)		0 (0.00)		17 (1.6)	
Mixed	71 (6.7)		0 (0.00)		71 (6.8)	
Other	44 (4.1)		2 (6.7)		42 (4.1)	
**LVSI**	331 (31.3)	28	3 (10.7)	1030	328 (31.8)	***0.017***^***c***^
**Tumor size (cm)**	3.0 [1.1, 4.5]	19	2.0 [0.60, 2.7]	1034	3.0 [1.1, 4.5]	***0.005***^***b***^
**Depth of myometrial invasion**		27		964		***0.004***^***c***^
0%	272 (27.4)		15 (55.6)		257 (26.7)	
< or = 50%	485 (48.9)		9 (33.3)		476 (49.4)	
>50%	234 (23.6)		3 (11.1)		231 (24.0)	
**Adjuvant therapy**	517 (48.3)	29	7 (24.1)	1042	510 (48.9)	***0.008***^***c***^
Chemotherapy	324 (30.2)	30	4 (13.3)	1042	320 (30.7)	***0.041***^***c***^
External beam	189 (17.6)	30	2 (6.7)	1042	187 (17.9)	0.11^c^
Vaginal brachytherapy	346 (32.3)	30	3 (10.0)	1042	343 (32.9)	***0.008***^***c***^

Statistics presented as median [P25, P75], *N* (column %)

*p* values: *b* Wilcoxon rank sum test, *c* Pearson’s chi-square test, *d* Fisher’s exact test

## Discussion

Mortality rates for EC have increased by 1.8% overall and 2.7% for non-endometrioid subtypes in recent years [14]. With hysterectomy being one of the most common gynecologic procedures performed, the importance of preoperative evaluation for malignancy is of utmost importance. The Federal Drug Administration warning against the use of power morcellation due to possible dissemination of occult malignancy has made for more robust preoperative cancer screening practices in patients with large uteri or abnormal uterine bleeding [15, 16]. Despite increased screening efforts, occult uterine malignancy is still identified in patients undergoing benign surgery. The incidence of occult EC was 0.56% in our study, which is consistent with previously reported data. In our study, patients with occult EC were more likely to have FIGO grade 1 disease, and less likely to have LVSI, larger tumor size, and deep MMI than the preoperatively diagnosed cohort. Furthermore, occult patients had a significantly lower body mass index and were less likely to be of African American race than the preoperatively diagnosed cohort. Overall, 83.3% (*n* = 25) patients received care in compliance with NCCN guidelines for uterine cancer.

Given recent increases in rates of hysteropexy [17], awareness of risk factors for occult endometrial pathology is important. While the literature does not consistently delineate risk factors, as outlined above, there are established contraindications to uterine preservation. Patients with genetic predisposition for malignancy and those with a personal history of an estrogen receptor positive breast cancer, especially if on tamoxifen, should not be offered uterine preserving prolapse repair. Other contraindications include endometrial hyperplasia and post-menopausal bleeding, even if the endometrial biopsy is negative. Obesity is cited as a relative contraindication to uterine preservation, given that this is a risk factor for development of endometrial cancer [18].

Identifying and understanding risk factors for incidental uterine pathology at time of hysterectomy is essential for optimizing preoperative screening and patient counseling in women undergoing hysterectomy for benign indications. Several studies have reviewed this topic. Reported rates of incidental EC or hyperplasia range from 0.3 to 2.6% [8, 9, 19]. Some notable risk factors in these studies include age >60 years, a concomitant diagnosis of hypertension or diabetes, and uterine weight > 250 grams. However, these factors were not found to be predictive of occult EC in our cohort [19]. In our study, the occult cohort had significantly different body mass index, race, and smoking history compared to the preoperatively diagnosed cohort. Previously published occult EC rates after surgery for varying benign indications were consistent with our findings.

Our study is also one of the few to report and compare oncologic descriptors and treatment outcomes after an occult EC diagnosis, and compare these women to a similar cohort of patients with preoperatively diagnosed EC. The incidental diagnosis of EC after surgery creates a dilemma in the absence of prognostic information that lymph node status provides [13]. In our study, ten patients (33.3%) underwent a second staging procedure and in eight (26.7%) patients, a second staging procedure was documented as discussed with the provider in the medical record, but was deferred due to patient preference or low risk status. Ambiguity regarding adjuvant care in situations when EC is identified on pathology specimens can be mitigated by the use of clinical criteria to estimate risk of recurrence and lymph node metastasis or use of a risk scoring system that has been described previously [20]. These tools can allow patients to receive the most appropriate surgery or adjuvant therapy for their cancer, and optimize oncologic outcomes even when cancer is incidentally diagnosed. Additionally, NCCN provides guidelines for patients with incidentally diagnosed EC who are incompletely staged which may include observation, vaginal brachytherapy, surgical restaging, or further imaging with treatment decisions altered on the basis of positive or suspicious imaging results [13].

Less than 25% of women in our study required adjuvant therapy. The majority of our patients had early-stage and low-grade disease and thus were able to be safely and appropriately observed. Emerging data regarding molecular classification of EC and its prognostic role may further simplify risk stratification and support evidence-based treatment decisions according to molecular subtypes [21]. These criteria are anticipated to enhance oncologic care, and allow women to avoid undertreatment or overtreatment of their disease.

The incidental diagnosis of EC is very low in patients undergoing hysterectomy for benign indications, with varying estimates ranging from 0.5–2.6% [8–10]. The incidental finding of uterine malignancy at time of hysterectomy for prolapse repair highlights continued difficulty with preoperative screening and diagnosis of EC, as most patients in this population were asymptomatic and had no identifiable risk factors. Despite the excellent outcomes and diagnosis of early-stage low-grade disease, an incidental diagnosis of EC is alarming to patients. The lifetime risk of an American woman developing EC is approximately 2.8%, and rising in recent years as previously described [22]. Our study contributes to the published literature, in providing support that the incidence of incidentally diagnosed EC at the time of hysterectomy for POP is lower than a woman’s lifetime risk of developing the disease. Future research is needed, especially in regard to cost effectiveness of preoperative screening with transvaginal ultrasound or endometrial biopsy in the setting of low risk patients and a rare primary outcome of incidentally diagnosed EC at time of surgery for benign indications.

The main strength of our study is that it includes data from a large volume academic institution with specialized gynecologic pathology review. Only two reviewers performed the chart review to minimize selection bias and standardize data collection. In addition, we were able to conduct a comparison of oncologic parameters to a similar cohort at the same institution with known EC at the time of primary surgery.

There are several limitations to our study that must be considered. The retrospective study design, the possibility of bias in patient selection by different staff surgeons, and the availability of medical records data cannot be disregarded. Furthermore, with the small sample size of the occult EC cohort and overall low incidence of postoperative complications, it is possible that the study was not adequately powered to detect significance with respect to specific associations. The patients in this study were treated at a high-volume tertiary care center and the majority identified as white, which may limit generalizability. The study also occurred over a 10-year study period with discrepancy in duration of study between the occult and preoperatively diagnosed cohort due to data availability, which may not account for practice or societal guidelines changes.

In summary, in this single institution retrospective cohort study of women incidentally diagnosed with EC at time of POP repair, the incidence of EC was similar to published rates in the literature. The majority of our patients received care concurrent with NCCN recommendations for EC despite their incidental diagnosis.

## Abbreviations

EC: Endometrial cancer
POP: Pelvic organ prolapse
CPT: Current Procedural Terminology
NCCN: National Comprehensive Cancer Network
LVSI: Lymphovascular space invasion
MMI: Myometrial invasion
FIGO: International Federation of Gynecology and Obstetrics
